# Antiangiogenic drugs in combination with seaweed fucoidan: A mechanistic *in vitro* and *in vivo* study exploring the VEGF receptor and its downstream signaling molecules in hepatic cancer

**DOI:** 10.3389/fphar.2023.1108992

**Published:** 2023-02-17

**Authors:** Maha R. A. Abdollah, Aya A. Ali, Hassnaa H. Elgohary, Mohamed M. Elmazar

**Affiliations:** ^1^ Department of Pharmacology, Faculty of Pharmacy, The British University in Egypt, El Sherouk City, Egypt; ^2^ Center for Drug Research and Development (CDRD), Faculty of Pharmacy, The British University in Egypt, El Sherouk City, Egypt

**Keywords:** angiogenesis, fucoidan, sorafenib, avastin, hepatocellular carcinoma, VEGF, VEGFR

## Abstract

Hepatocellular carcinoma (HCC) is one of the most common cancers reported worldwide with poor morbidity and high mortality rates. HCC is a very vascular solid tumour as angiogenesis is not only a key driver for tumour progression but also an exciting therapeutic target. Our research investigated the use of fucoidan, a sulfated polysaccharide readily abundant in edible seaweeds commonly consumed in Asian diet due to their extensive health benefits. Fucoidan was reported to possess a strong anti-cancer activity, but its anti-angiogenic potential is still to be fully unraveled. Our research investigated fucoidan in combination with sorafenib (an anti-VEGFR tyrosine kinase inhibitor) and Avastin^®^ (bevacizumab, an anti-VEGF monoclonal antibody) in HCC both *in vitro* and *in vivo*. *In vitro* on HUH-7 cells, fucoidan had a potent synergistic effect when combined with the anti-angiogenic drugs and significantly reduced HUH-7 cell viability in a dose dependent manner. Using the scratch wound assay to test cancer cell motility, sorafenib, A + F (Avastin and fucoidan) or S + F (sorafenib and fucoidan) treated cells consistently showed an unhealed wound and a significantly smaller %wound closure (50%–70%) *versus* untreated control (91%–100%) (*p* < 0.05, one-way ANOVA). Using RT-qPCR; fucoidan, sorafenib, A + F and S + F significantly reduced the expression of the pro-angiogenic PI3K/AKT/mTOR and KRAS/BRAF/MAPK pathways by up to 3 folds (*p* < 0.05, one-way ANOVA *versus* untreated control). While ELISA results revealed that in fucoidan, sorafenib, A + F and S + F treated cells, the protein levels of caspases 3, 8, and 9 was significantly increased especially in the S + F group showing 40- and 16-times higher caspase 3 and 8 protein levels, respectively (*p* < 0.05, one-way-ANOVA *versus* untreated control). Finally, in a DEN-HCC rat model, H&E staining revealed larger sections of apoptosis and necrosis in the tumour nodules of rats treated with the combination therapies and immunohistochemical analysis of the apoptotic marker caspase 3, the proliferation marker Ki67 and the marker for angiogenesis CD34 showed significant improvements when the combination therapies were used. Despite the promising findings reported herein that highlighted a promising chemomodulatory effect of fucoidan when combined with sorafenib and Avastin, further investigations are required to elucidate potential beneficial or adversary interactions between the tested agents.

## 1 Introduction

Hepatocellular carcinoma (HCC) is the third most common cause of cancer mortality and thus considered a major global health problem (El-Serag and Rudolph, 2007; Tawfik et al., 2022). Eighty percent of HCC cases are reported in the developing countries in East Asia and sub-Saharan Africa representing a major economic burden (El-Serag and Rudolph, 2007; Abdelmageed et al., 2016).

HCC is a highly vascular tumour and therefore represents an attractive candidate for the development of anti-angiogenic drugs (Sampat and O'Neil, 2013; Morse et al., 2019). The process by which new blood vessels are formed from existing ones is called angiogenesis, one of the main hallmarks of cancer (Hanahan and Weinberg, 2011) and a key tumorigenic driver responsible for delivering the essential nutrients and oxygen needed by the tumour to continue its uncontrolled proliferation as well as the removal of unwanted metabolic waste (Nishida et al., 2006). The ability of cancer cells to travel to distant organs *via* the newly formed blood vessels, i.e. metastasis, is one of the inherent dangers associated with angiogenesis as well as one of the main causes of mortality from cancer (Nishida et al., 2006). Therefore, both tumorigenesis and metastatic spread rely mainly on angiogenesis which is triggered by a variety of angiogenic factors secreted from tumor cells (Sampat and O'Neil, 2013).

The main goal of anti-angiogenic therapies is to turn cancer into a “dormant” disease by cutting off the blood supply thus “starving” tumour cells to death. Despite years of research dedicated to developing potent therapies to halt angiogenesis, the full therapeutic potential of anti-angiogenic drugs is yet to be fulfilled. Modest overall survival benefits accompanied with the emergence of resistance are few of the challenges that stop anti-angiogenic drugs from reaching their full therapeutic potential (Yang et al., 2017). Sorafenib, an oral multi-kinase inhibitor, and Avastin, an anti-VEGF monoclonal antibody, are two anti-angiogenic agents currently approved for treating unresectable or metastatic HCC (Morse et al., 2019). Unfortunately, clinical data revealed patients’ non-compliance caused by severe side effects, toxicity and tumour relapse (Bonam et al., 2018). This highlights the urgency to develop adjuvant new agents to improve the therapeutic outcome of anti-angiogenic agents.

Medicinal plant-based herbal products have gained a lot of momentum in pursue of safer as well as more efficient anti-cancer therapies. They are mainly used as adjuvant therapies to reduce the toxic side effect of standard chemotherapies (Atashrazm et al., 2015; Bonam et al., 2018; Mahmoud et al., 2022). Natural polysaccharides obtained from algae and marine plants have drawn a lot of attention in recent years owing to their potential anti-cancer effect as well as their diverse biological activities (Atashrazm et al., 2015). Of these polysaccharides, fucoidan (a natural seaweed extract) has been under intense research due to its versatile biological activities (Fitton, 2011). Some of the reported biological activities of fucoidan include antiviral, anticoagulant, anti-inflammatory, antioxidant and antihyperlipidemic (Cumashi et al., 2007; Li et al., 2008; Lira et al., 2011; Abdollah et al., 2018). Fucoidan was also evaluated for potential therapeutic action in liver and kidney diseases, osteoarthritis and stem cell modulation (Fitton, 2011).

Fucoidan is a sulphated polysaccharide consisting mainly of fucose and sulfate ester groups (Cumashi et al., 2007; Li et al., 2008). It is extracted from different species of brown seaweed (e.g., *Laminaria japonica*, *Undaria pinnatifida*, *Fucus vesiculosus*, and *Macrocystis pyrifera*) and some marine invertebrates (e.g., sea urchins and sea cucumbers) (Li et al., 2008; Fitton, 2011; Atashrazm et al., 2015). In addition to being consumed in traditional Asian diet (particularly in China, Korea and Japan), it is commercially available as an over the counter (OTC) herbal food supplement in many western countries (Fitton, 2011; Atashrazm et al., 2015). Therefore, it is considered an exciting agent for clinical development owing to its relative safety and bioavailability.

The diverse pharmacological actions of fucoidan are still under investigation but it has been reported to affect many pathophysiological processes, such angiogenesis, carcinogenesis, oxidative stress, immune modulation and inflammation (Kwak, 2014; Jin et al., 2022). Both *in vitro* and *in vivo* studies have consistently confirmed the anti-cancer potential of fucoidan *via* the inhibition of angiogenesis, induction of cell cycle arrest and apoptosis (Aisa et al., 2005; Zhang et al., 2011; Atashrazm et al., 2015) and down regulation of CDK4, cyclin D1 and cyclin D2 in cancer cells (Banafa et al., 2013; Boo et al., 2013) [reviewed in more details in (Atashrazm et al., 2015) and (Jin et al., 2022)]. Fucoidan also modulated a number of oncogenic signaling pathways known to be upregulated in cancer and involved in promoting tumour progression, for instance, the extracellular signal-regulated kinase (ERK) pathway (or RAS/RAF/MAPK pathway) (Aisa et al., 2005), PI3K/AKT/mTOR pathway (Lee et al., 2012) as well as the GSK and Wnt pathways (Boo et al., 2013).

**FIGURE 1:**
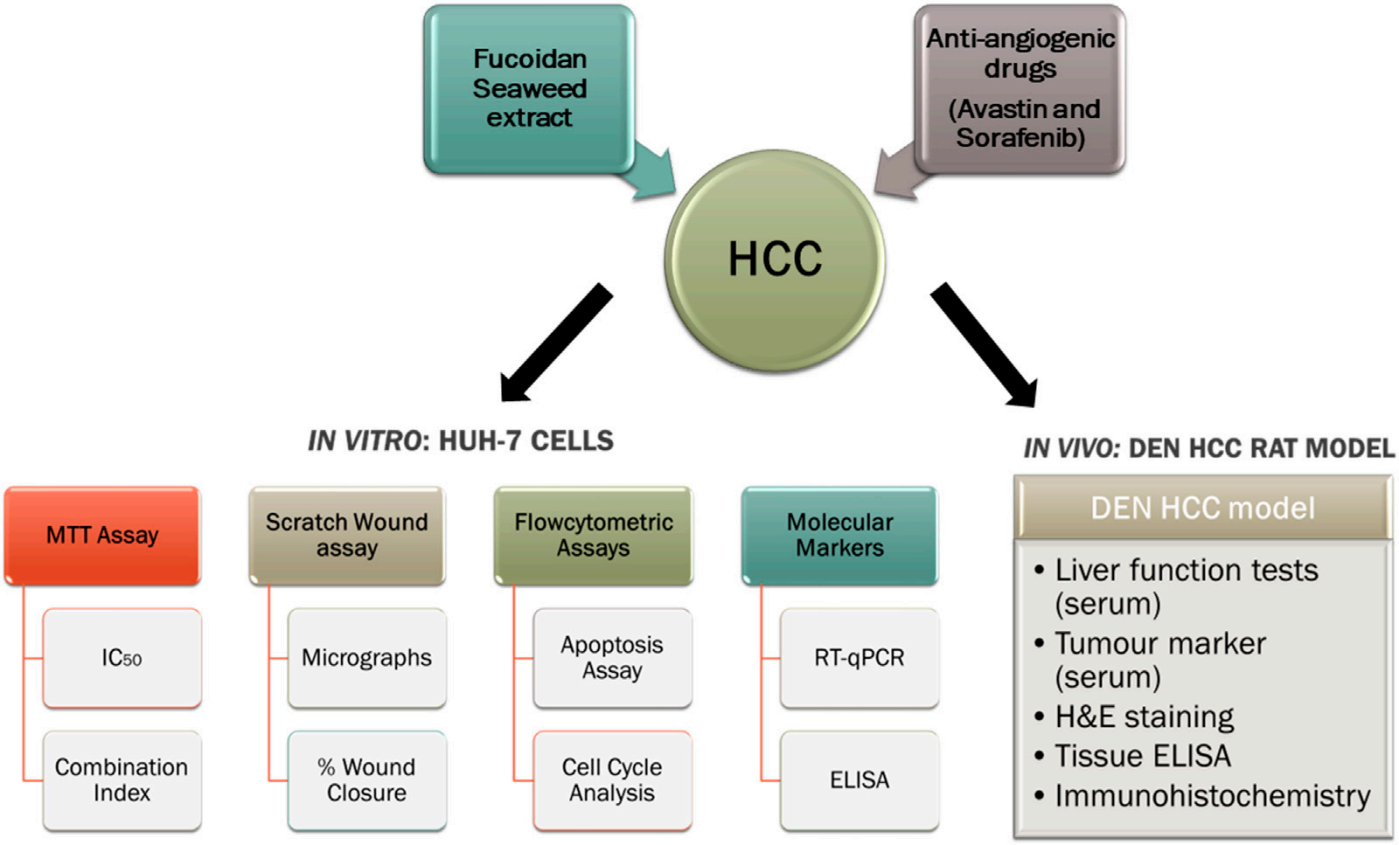
A graphical summary of our experimental design.

The aim of our study was to investigate the chemomodulatory effect of fucoidan, as an adjuvant therapy, in combination with two FDA-approved antiangiogenic agents: sorafenib and Avastin (bevacizumab) to potentiate their pharmacological action and potentially reduce their toxic side effects. A limited number of anti-angiogenic agents demonstrated efficacy in the treatment of advanced HCC despite decades of research (Raoul et al., 2017). Furthermore, very limited studies have reported the combination of anti-angiogenic agents with fucoidan, thus, our research might help better elucidate the interaction of fucoidan with the angiogenic pathways and unravel the potential molecular pathways and pharmacological mechanisms involved. A graphical abstract explaining our experimental approach is shown in Figure 1.

## 2 Materials and methods

### 2.1 Materials

Human hepatocellular carcinoma cell line HUH-7 was purchased from Vacsera (Giza, Egypt), sorafenib p- Toluenesulfonate salt was purchased from LC Laboratories (Woburn, MA, United States), Avastin^®^ (bevacizumab, Genentech, United States) was purchased, in its formulated commercial preparation, from a community Pharmacy (Cairo, Egypt) and fucoidan extracted from *Laminaria Japonica* was purchased as a crude extract from Buchem BV (Holland). Dulbecco’s modified Eagle’s medium (DMEM) medium and fetal bovine serum (FBS) were purchased from Gibco^®^, Thermo Fisher Scientific, United States. Antibiotic-antimycotic mixture (100 U/ml of penicillin, 0.1 U/ml streptomycin and 0.25 μg/ml of Amphotericin B) was purchased from Lonza^®^, Walkersville, MD, United States. All other chemicals were purchased from Sigma Aldrich, United States unless otherwise specified.

### 2.2 Cell culture

HUH-7 cells were maintained in culture media consisting of DMEM supplemented with 10% fetal bovine serum (FBS), 100 U/ml of penicillin, 0.1 U/ml streptomycin, 0.25 μg/ml of Amphotericin B at 37°C in a humidified incubator containing 5% CO_2_. All cell culture procedures were performed in a class II laminar flow hood and incubations were done inside the incubator at 37°C, unless otherwise specified.

### 2.3 Cell viability (MTT) assay

Cytotoxicity was evaluated using the MTT (3-(4,5-Dimethylthiazol-2-yl)-2,5-diphenyltetrazolium bromide) cell viability assay. 20,000 cells/well were seeded in a 96-well plate and left to attach overnight (o/n). Next day, the cells were treated with increasing concentrations of Avastin (0.94–30 µM), sorafenib (0.3–20 µM) and fucoidan (0.15—5 mg/ml) for 72 h. Next, media was discarded and replaced with 100 μl/well of fresh DMEM containing 12 mM (5 mg/ml) MTT stock in complete media and further incubated for 2 h inside the incubator at 37°C. Finally, the media was discarded and replaced with 100 μl of DMSO to dissolve the formed formazan crystals and then the absorbance was measured at 570 nm. Inhibitory concentration 50 (IC_50_) was calculated using GraphPad Prism Software using the non-linear regression analysis.

For combination therapies, cells were seeded as previously described then next day treated with increasing concentrations of Avastin (0.94–30 µM), sorafenib (0.3–20 µM) either alone or in combination with IC_3_ of fucoidan (55 μg/ml) for 72 h. The pharmacological interaction between fucoidan and sorafenib or Avastin when used in combination was evaluated by applying the isobologram equation shown below to calculate the combination indices (Chou, 2006).

$$Combination\;index\;\left(CI\right)=\left(D\right)1\;/\left(D_{n}\right)1+\left(D\right)2/\left(D_{n}\right)2$$

Where (D_n_)1 and (D_n_)2 are the concentrations of each drug alone to exert n% effect, while (D)1 and (D)2 are the drug concentrations when used in combination to exert the same effect. The pharmacological interaction of the combination is estimated to be synergistic if CI < 0.8; additive if CI ranges from 0.8 to 1.264 or antagonistic if CI > 1.2.

### 2.4 Scratch wound assay

Briefly, 10^6^ HUH-7 cells were seeded in six 6-well plates and allowed to attach overnight (o/n). Once cells reached confluency, the cell monolayer was scratched using a 200 μL pipette tip held vertically followed by washing twice with PBS to remove floating cells. The cells in each plate were then treated with complete DMEM medium alone (untreated control) or added to it either 5 µM sorafenib, 25.22 µM Avastin, 55 μg/ml fucoidan or the combinations of sorafenib and fucoidan (S + F) or Avastin and fucoidan (A + F). The wound area was imaged on day 0, 1, 2, 3 and 4 post treatment using an inverted microscope (Labomed Inc., LA, CA, United States) connected to a digital camera. Wound width was calculated using a wound healing size plugin for ImageJ^®^ (NIH, United States) as described by (Suarez-Arnedo et al., 2020).

### 2.5 Annexin V/propidium iodide (PI) apoptosis assay on HUH-7 cells

HUH-7 cells were seeded in T-25 flasks and allowed to attached o/n. Next day, cells were treated with 5 µM sorafenib, 25.22 µM Avastin, 55 μg/ml fucoidan or the combinations of sorafenib and fucoidan (S + F) or Avastin and fucoidan (A + F) for 72 h. Next, cells were detached using trypsin, rinsed with cold 1x PBS, centrifuged twice at 280 x g for 5 min, resuspended in cold PBS and kept on ice until analysis. To a 100 µl aliquot of each cell suspension, 1 µl of PI stock (prepared at 100 μg/ml) and 5 µl Annexin V-FITC were added for 15 min at room temperature in the dark. Finally, 400 µl of 1x Annexin binding buffer was added to each sample and analyzed by CytoFlex flow cytometer (Beckman Coulter, CA, United States) according to the manufacturers’ instructions. A minimum of 10,000 events were recorded for each sample. Data analysis was done using FlowJo software (Treestar Inc., San Carlos, CA, United States).

### 2.6 Cell cycle analysis

Cells were grown in six 6-well plates and each plate was subsequently treated with either 5 µM sorafenib, 25.22 µM Avastin, 55 μg/ml fucoidan or the combinations of sorafenib and fucoidan (S + F) or Avastin and fucoidan (A + F) for 48 h. Next, cells were harvested, washed with cold 1x PBS, centrifuged twice at 280 x g for 5 min and resuspended in cold PBS and kept on ice until analysis. Cell pellets were fixed by being re-suspended in 2 ml of 60% ice-cold ethanol at 4°C for 1 h. Fixed cells were subsequently washed at least twice with PBS (pH 7.4). The cell pellet was then re-suspended in 1 ml of nuclei acid staining mixture (10 μg/ml propidium iodide (PI) and 50 μg/ml RNAase A in PBS) for 20 min in the dark at 37°C. Cells were analyzed for DNA contents using flow cytometry analysis using CytoFlex flow cytometer (Beckman Coulter, CA, United States) according to the manufacturers’ instructions. For each sample, 10,000 events were acquired. Cell cycle distribution was calculated using CytExpert software (Beckman Coulter, CA, United States).

### 2.7 Real time quantitative polymerase chain rection (RT-qPCR)

HUH-7 cells were seeded in T-75 flasks overnight at a seeding density of 5 × 10^6^ cells/flask. Next day, the media was discarded and replaced with DMEM medium alone (untreated control) or added to it either 5 µM sorafenib, 25.22 µM Avastin, 55 μg/ml fucoidan or the combinations of sorafenib and fucoidan (S + F) or Avastin and fucoidan (A + F) and the cells were incubated for 72 h. Next, total RNA was isolated using RNeasy^®^ Kits (Qiagen, Hilden, Germany) according to the manufacturer’s protocol. RNA samples were then assessed to detect purity by measuring the absorbance of the RNA samples using NanoDrop Spectrophotometer (BMG LABTECH, Ortenberg, Germany) at 260 nm (ng/μl) and calculating the A260/280 ratio. cDNA was synthesized using the Revertaid cDNA synthesis kit (K1621; Thermo Fisher Scientific, MA, United States), according to the manufacturer’s instructions. Primers used were purchased from Thermo Fisher (MA, United States). Gene expression levels were calculated as follows: 2^−ΔΔCT^ ± standard deviation. Full list of primers’ sequences is shown in Table 1.

**TABLE 1:** List of primers’ sequences used in the RT-qPCR experiments.

Gene	Forward primer	Reverse primer
PI3K	ACCTTGTTCCAATCCCAGGT	TCGGCCTTTAACAGAGCAAA
AKT1	TATGGCGCTGAGATTGTGTC	AAAGGTCTTCATGGTGGCAC
mTOR	CCCTACTTTGCTTGAGGTGC	TGGATTCTGACAGGCTGACA
KRAS	TACAGTGCAATGAGGGACCA	TCCTGAGCCTGTTTTGTGTC
BRAF	ATTTGGGCAACGAGACCGAT	GTTGATCCTCCATCACCACGA
MAPK1	CCCCATCACAAGAAGACCTG	CTCGTCACTCGGGTCGTAAT
β-actin	AGCACAGAGCCTCGCCTTT	CACGATGGAGGGGAAGAC

### 2.8 Protein extraction for ELISA

HUH-7 cells were seeded in T-75 flasks overnight at a seeding density of 5 × 10^6^ cells/flask. Next day, the media was discarded and replaced with DMEM medium alone (untreated control) or added to it either 5 µM sorafenib, 25.22 µM Avastin, 55 μg/ml fucoidan or the combinations of sorafenib and fucoidan (S + F) or Avastin and fucoidan (A + F) and the cells were incubated for 72 h. Next, cells were collected *via* trypsinization and then washed twice with cold PBS and re-pelleted. The cell pellet was resuspended in 100 μl cell lysis buffer (1 ml of RIPA lysis buffer (Thermo Scientific, United States, Catalogue number: 89,900) + 10 µl of HALT™ protease inhibitor (Thermo Scientific, United States, Catalogue number: 78,410) + 10 µl of HALT™ phosphatase inhibitor (Thermo Scientific, Catalogue number: 78,420) and incubated for 30 min on a rotor at 4°C. Next, the samples were centrifuged at 280 x g for 10 min at 4°C and the supernatants were transferred to fresh tubes and placed on ice. Total protein concentration was measured using BCA protein assay as per the manufacturer’s protocol (Thermo Scientific, United States). The samples were stored at −80°C until processing.

### 2.9 VEGF, caspase 3, 8, and 9 quantification in whole cell lysates using ELISA

Total protein was quantified in whole cell lysates as described above; all samples were diluted with dH_2_O to contain 1 mg/ml total protein. 80 µl of each sample (whole cell lysate) was used in each ELISA and the manufacturer’s protocol was followed. All ELISA kits used were from Invitrogen, Thermo Scientific, United States as follows: human VEGF ELISA kit (catalogue number KHG0111); human caspase 3 (active) ELISA kit (catalogue number KHO1091), human caspase 8 ELISA kit (catalogue number BMS2024) and human caspase 9 ELISA kit (catalogue number BMS2025).

### 2.10 Diethyl nitrosamine (DEN) HCC rat model

All animal procedures met the Animals (Scientific Procedures) Act 1986/ASPA Amendment Regulations 2012 and were done after the approval of the Ethical Review committee at the British University in Egypt (Ethics approval number: EX-2218).

46 Sprague Dawley male rats, 5–6 weeks old (100–150 g) were injected intraperitonially (I.P) with 50 mg/kg DEN dissolved in saline once a week for 16 weeks (Schiffer et al., 2005). At the beginning of the 17^th^ week, rats were randomly divided into 6 groups (n = 7–8): Group 1: Untreated control, group 2: Received 200 µg per rat Avastin I.P once per week. Group 3: Received sorafenib at 10 mg/kg *via* oral gavage once per day for five consecutive days. Group 4: Received fucoidan at 20 mg/kg I.P once per day for five consecutive days while Group 5 received the combination of Avastin and fucoidan and Group 6 received the combination of sorafenib and fucoidan. Treatments were continued for 4 weeks then mice were sacrificed by cervical dislocations. Blood and tissue samples were collected and stored appropriately until further analysis.

### 2.11 Measuring serum levels of ALT, AST, and AFP

Alanine aminotransferase (ALT) and aspartate aminotransferase (AST) were assessed in the serum samples taken from rats using colorimetric commercial kits from Spectrum diagnostics, Cairo, Egypt as per the manufacturer’s protocol. Serum alpha fetoprotein (AFP) was measured using rat AFP ELISA kit (Elabscience, United States, catalogue number E-EL-R01047) as per the manufacturer’s protocol.

### 2.12 Histopathological analysis of tissues extracted from DEN HCC model

For histopathological examination, liver tissues were preserved in 10% formalin solution for at least 48 h. Samples were then rinsed with tap water and treated with serial dilutions of methanol, ethanol and absolute ethanol for tissue dehydration followed by xylene. Tissue samples were embedded in paraffin wax and kept at 56°C for 24 h in a hot air oven. Next, the tissue blocks were sliced into 4 μm thick sections and placed on glass slides, deparaffinized, subsequently stained with hematoxylin & eosin (H&E) and examined with light microscopy (Banchroft et al., 1996).

### 2.13 Measuring the levels of VEGF, VEGFR and AFP in liver tissues using ELISA

Liver tissue homogenates extracted from rats of the different treatment groups were prepared and evaluated for the levels of VEGF, VEGFR and AFP using ELISA kits as per the manufacturer’s protocol. ELISA kits used were rat VEGFR1/FLT1 ELISA kit (Elabscience, United States, catalogue number E-EL-R011108); rat VEGF ELISA kit (Creative Diagnostics, United States, catalogue number DEIA1173) and rat AFP ELISA kit (Elabscience, United States, catalogue number E-EL-R01047).

### 2.14 Immunohistochemistry analysis of CD34, caspase 3 and Ki67

Avidin-Biotin immunoperoxidase complex technique (ABC) was used (Hsu et al., 1981). Briefly, paraffin embedded tissues were deparaffinized and rehydrated through graded alcohol series. Antigen retrieval was done by incubating tissue sections in an antigen retrieval citrate buffer in a microwave oven for 5 min at 700 w, then sections were left to cool. To quench endogenous peroxidase activity, 2 drops of peroxidase blocking serum was added for 10 min, then slides were rinsed with PBS (pH 7.4). Next, two drops of protein blocking serum were added for 10 min followed by incubation with either of the following primary antibodies: caspase-3 rabbit polyclonal antibody (catalogue number A2156, ABclonal, Woburn, MA, United States), CD34 rabbit monoclonal antibody (catalogue number A19015, ABclonal, Woburn, MA, United States) or Ki67 rabbit monoclonal antibody (Clone QR015, Quartett, Berlin, Germany) for 30 min at room temperature. Next, pre-diluted biotinylated secondary antibody was added to each section for 45 min followed by rinsing with PBS. Horseradish peroxidase conjugated streptavidin was added for 20 min followed by rinsing with PBS. Substrate/chromogen (DAB) mixture (which was prepared immediately before use) was added and slides were incubated for 5–10 min followed by rinsing with dH_2_O. Finally counterstaining was done with Harris’s hematoxylin and sections were dehydrated with graded alcohol series, cleared in xylene, and finally mounted by DPX. Sections were imaged using Leica Application software for tissue sections analysis (Leica Microsystems GmbH, Germany).

### 2.15 Statistical analysis

Experimental results were plotted as mean values ±standard error of mean (SEM) unless otherwise specified. Statistical analysis for multiple experimental groups was done using one-way analysis of variance (ANOVA) followed by *post hoc* Tukey-Kramer test (unless otherwise specified) with *p* values considered statistically significant if less than or equal 0.05. All statistical analyses and data plotting were done using GraphPad Prism software, version 5.00 (GraphPad Software, Inc. La Jolla, CA, United States). All experiments were repeated at least 3 times with 3-6 replicates per treatment. Representative data is shown.

## 3 Results

### 3.1 Cell viability (MTT) assay

**FIGURE 2:**
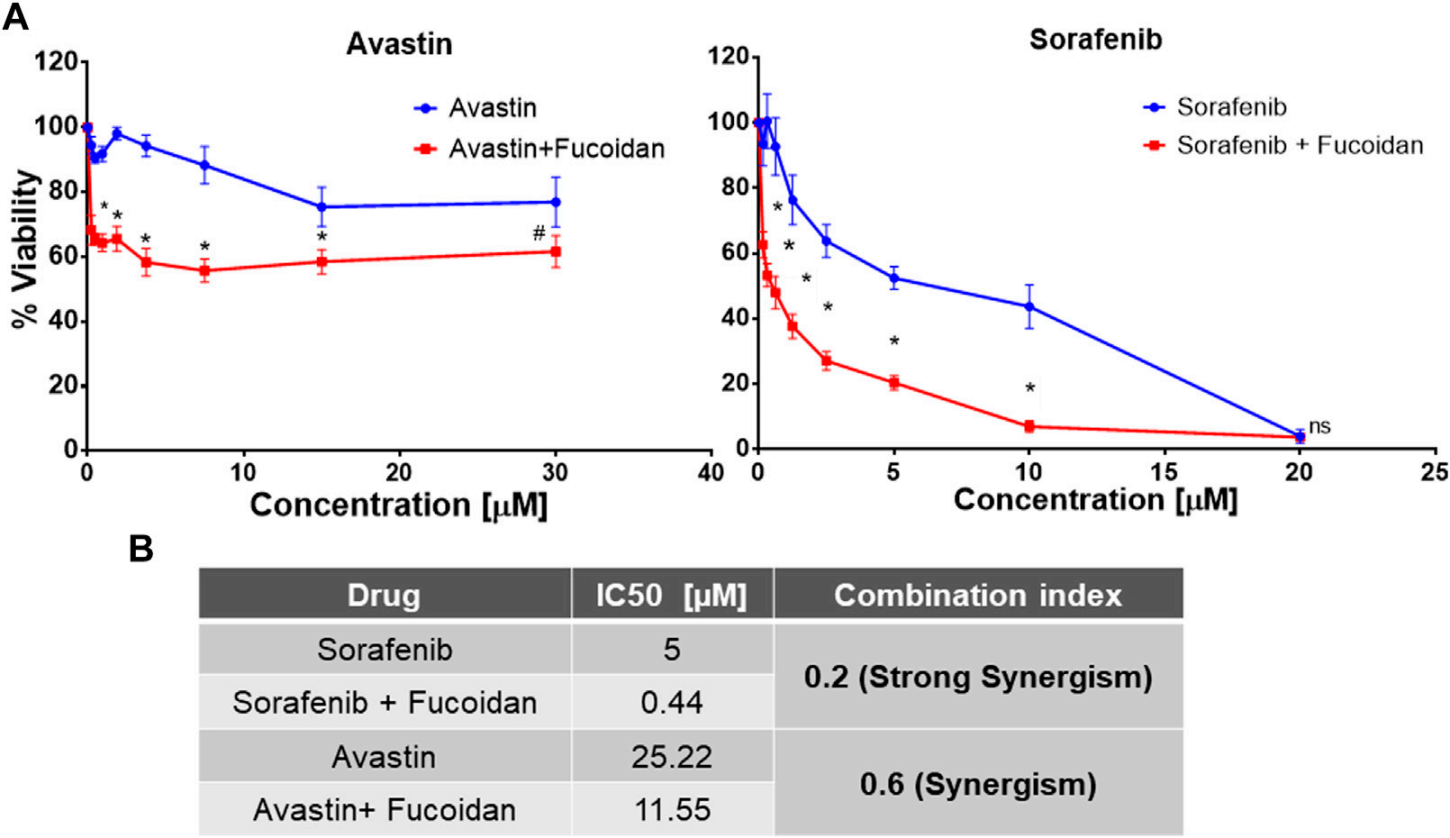
**(A)** MTT cell viability assay after 72 h of incubating HUH-7 cells with either sorafenib or Avastin monotherapies or in combination with the IC_3_ of fucoidan (55 μg/ml) (n = 6 per treatment, experiment was repeated at least 4 times, *p < 0.0001 and #*p* = 0.002, Student’s two-tailed T-test *versus* monotherapies). **(B)** Summary table showing the calculated IC_50_ of each drug alone or in combination with IC_3_ of fucoidan (55 μg/ml) as well as the combination indices indicating a synergistic interaction between fucoidan and the 2 anti-angiogenic drugs.

HUH-7 cells were treated with various concentrations of Avastin, sorafenib and fucoidan as single therapies for 72 h. Inhibitory concentrations 50 (IC_50_) were calculated for each drug individually (data not shown). Next, fucoidan was combined with the anti-angiogenic drugs and results showed a reduction in the IC_50_ of sorafenib from 4.9 to 0.4439 µM and Avastin from 25.22 to 11.55 µM when combined with fucoidan with a calculated combination indices of 0.2 and 0.6, respectively. These findings suggest a synergistic interaction between the anti-angiogenic drugs and fucoidan (Chou, 2006). (As shown in Figure 2)

### 3.2 Scratch wound assay

The ability of sorafenib, Avastin and fucoidan and their respective combinations to alter HUH-7 cells migration was analyzed *via* the scratch wound healing assay.

**FIGURE 3:**
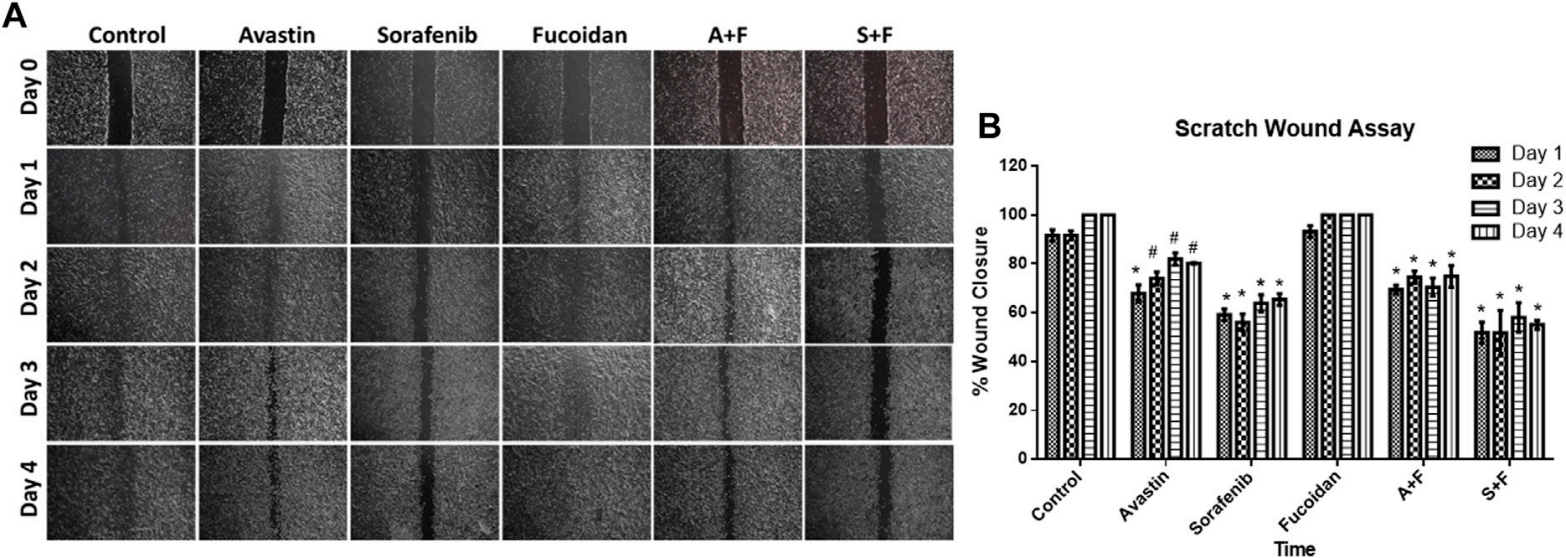
**(A)** Scratch wound assay showing photomicrographs of HUH-7 cells after 1–4 days of drug incubations. **(B)** Percentage wound closure calculated from the photomicrographs of the scratch wound assay using ImageJ. Sorafenib, A + F and S + F treated cells consistently showed an unhealed wound and a significantly smaller % wound closure (sorafenib: 59%–63%; A + F: 69%–70% and S + F: 51%–58%) throughout the incubation period *versus* the untreated control in which the wound almost completely healed (91%–100%) (n = 3 wells/treatment, #*p* < 0.05, *p < 0.001 *versus* untreated control at equal time points, two-way ANOVA, Tukey’s *post hoc*).

Throughout the span of the experiment (4 days); sorafenib, A + F and S + F treated cells consistently showed an unhealed wound indicating the inhibition of cancer cell proliferation and migration unlike the untreated control and fucoidan treated cells where the wound rapidly disappeared throughout the span of the experiment (Figure 3). While Avastin treated cells had a small wound remaining on day 1 and 2 with an almost completely healed wound on day 4. The group S + F followed by sorafenib then A + F showed the best results with almost a slightly smaller wound area observed on day 4 compared to day 0. Next, wound width was quantified using ImageJ software and a plugin designed by (Suarez-Arnedo et al., 2020). Sorafenib, A + F or S + F treated cells consistently showed a significantly smaller % wound closure (sorafenib: 59%–63%; A + F: 69%–70% and S + F: 51%–58%) throughout the incubation period *versus* the untreated control in which the wound almost completely healed (91%–100%) (*p* < 0.05, two-way-ANOVA, Tukey’s *post hoc*).

### 3.3 Annexin V/PI apoptosis assay

**FIGURE 4:**
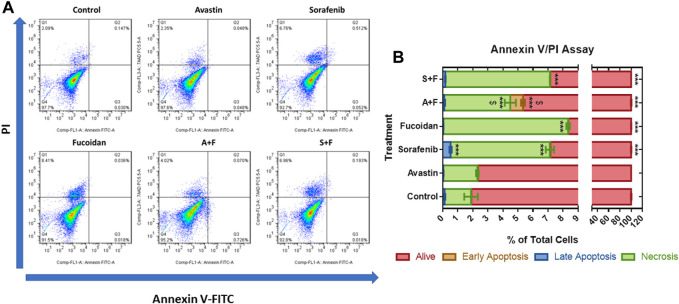
Annexin V/PI apoptosis assay showing the effect of Avastin, sorafenib, fucoidan monotherapies or in combination on the percentage of apoptosis in HUH-7 cells **(A)** Dot plots of Annexin V-FITC *versus* PI signal detected (representative data is shown). **(B)** Stacked bar chart showing the percentage of cells detected in each quadrant. 98.1% and 97.66% of the cell population was alive in the untreated control and Avastin with no significant difference between both groups. Sorafenib monotherapy caused a significant increase in the percentage of cells in late apoptosis (0.52%) and necrosis (6.6%) and a significant decrease in the percentage of live cells (92.8%) compared to untreated control. Fucoidan, A + F and S + F showed similar patterns with a significant decrease in live cells to 91.6%, 94.66% and 92.83%, respectively, with an increase of necrotic cells to 8.3%, 4.4% and 6.9%, respectively compared to untreated control. A + F was significantly better than Avastin monotherapy as higher percentage of cells were detected in early apoptosis and necrosis. While the S + F combination showed very similar findings to sorafenib monotherapy (n = 3 wells/treatment, ***p < 0.0001 *versus* untreated control and $*p* < 0.0001 compared to monotherapy, one-way ANOVA, Tukey’s *post hoc*).

To examine the role of apoptosis in the cytotoxic effect of the drugs, the percentage of apoptotic cells was detected *via* Annexin V/PI staining that was measured by flow cytometry. In the untreated control and Avastin, 98.1% and 97.66% of the cell population was alive with no significant difference between both groups. Sorafenib monotherapy caused a significant increase in the percentage of cells in late apoptosis (0.52%) and necrosis (6.6%) and a significant decrease in the percentage of live cells (92.8%) compared to untreated control (*p* < 0.0001). While fucoidan, A + F and S + F showed similar patterns with a significant decrease in live cells to 91.6%, 94.66%, and 92.83%, respectively (*p* < 0.0001) with an increase of necrotic cells to 8.3%, 4.4%, and 6.9%, respectively (*p* < 0.0001) compared to untreated control. The combination of A + F was significantly better than the Avastin monotherapy as it showed higher percentage of cells in early apoptosis and necrosis. While the S + F combination showed very similar findings to sorafenib monotherapy (Figure 4).

### 3.4 Cell cycle analysis

**FIGURE 5:**
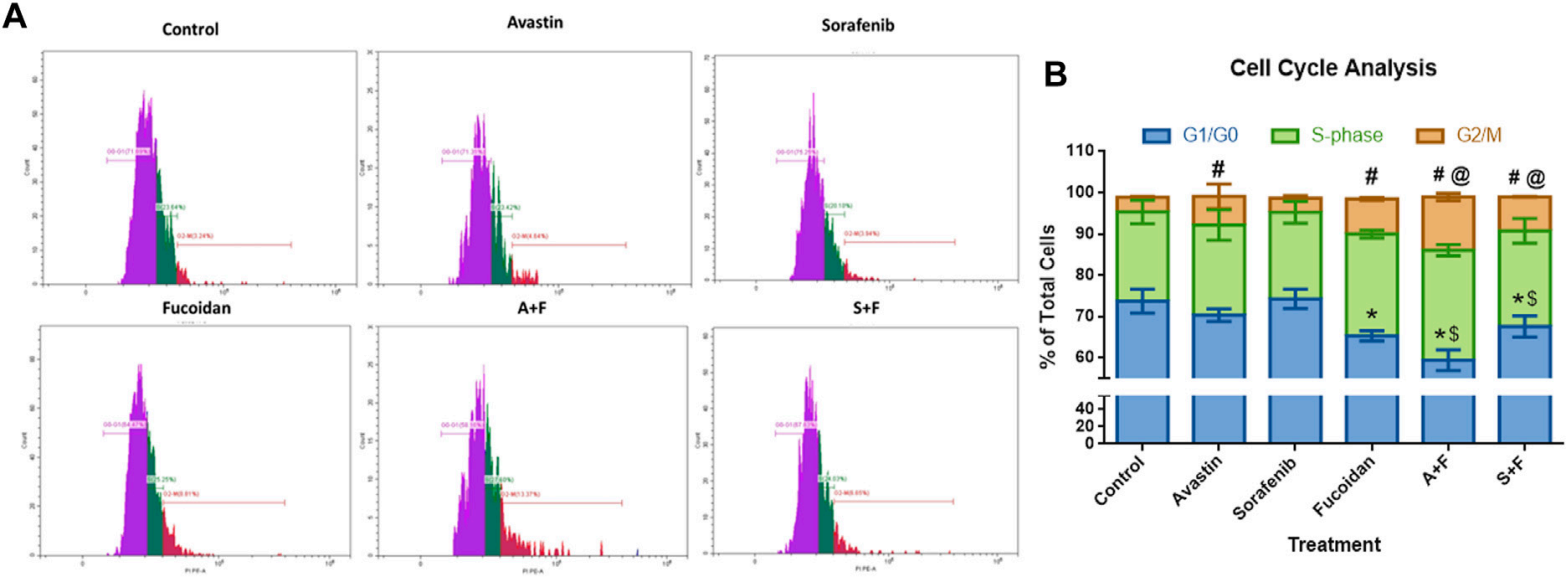
Cell cycle analysis showing the effect of Avastin, sorafenib and fucoidan as monotherapies or in combination on the cell cycle progression of HUH-7 cells. **(A)** Representative histograms showing the cell population distribution in each cell cycle phase after the various treatments. **(B)** Stacked bar chart showing the % of total cells detected in G0/G1, S-Phase and G2/M phases. No significant differences between the untreated control and sorafenib treated cells were observed with around 74% of the cells in the G0/G1 phase, 21% in S-phase and 3.5% in the G2/M phase. Avastin treated cells significantly had higher portion of cells arrested in the G2/M phase compared to the untreated control (7% versus 3.5%) while 70.4 % and 21.7% of the cells were detected in the G0/G1 and S-phases, respectively. In fucoidan, A+F and S+F treated cells, a different pattern was observed, with more cells arrested in the S-phase and G2/M phases compared to the untreated control. This pattern appears to be induced by fucoidan as both A+F and S+F had a significantly higher percentage of cells in the S-phase (26.6% and 23.1%, respectively) and G2/M phase (12.9% and 8.2%, respectively) versus the monotherapies. G0/G1: *p ≤ 0.01 versus untreated control and $ *p* <0.05 compared to monotherapy; G2/M: #*p* <0.05 versus untreated control and @ *p* <0.05 compared to monotherapy, One-way ANOVA, Tukey’s Post-Hoc.

We used this assay to examine the effect of the different drugs on the progression of the cell cycle. Results (Figure 5) revealed no significant differences between the untreated control and sorafenib treated cells with around 74% of the cells in the G0/G1 phase, 21% in S-phase and 3.5% in the G2/M phase. In the Avastin monotherapy group, significantly higher portion of cells were arrested in the G2/M phase compared to the untreated control (7% *versus* 3.5%, *p* < 0.05) while 70.4% and 21.7% were detected in the in G0/G1 and S-phases, respectively. In the fucoidan, A + F and S + F treated cells the pattern was different, with more cells arrested in the S-phase and G2/M phases compared to the untreated control. This pattern appears to be induced by fucoidan as both A + F and S + F had a significantly higher percentage of cells in the S-phase (26.6% and 23.1%, respectively) and G2/M phase (12.9% and 8.2%, respectively) *versus* the monotherapies (*p* < 0.05).

### 3.5 Effect of the combination therapy on the key apoptotic proteins using ELISA

**FIGURE 6:**
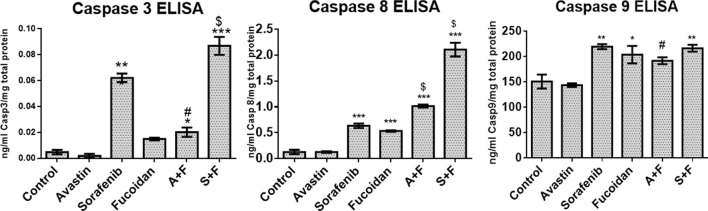
Caspase 3, 8, or 9 ELISA results done on HUH-7 whole cell lysates following treatment with Avastin, sorafenib and fucoidan as monotherapies or in combination. Results revealed that all drugs and combinations, except Avastin monotherapy, significantly increased the levels of caspases 3, 8, and 9 with the highest effect observed in the S + F group for caspase 3 and 8. (n = 3–4, *p < 0.05, **p < 0.005, ***p < 0.001 *versus* untreated control or ^$^ *p* ≤ 0.005 and ^#^ *p* < 0.05 compared to monotherapy, one-way ANOVA with Tukey’s *post hoc*).

The effect of the drugs was investigated on the three caspases that are the key players in both the intrinsic and extrinsic pathways of apoptosis (Van Opdenbosch and Lamkanfi, 2019). ELISA results (Figure 6) revealed that all drugs and their combinations, except Avastin monotherapy, significantly increased caspases 3 and 8 with S + F group showing 40- and 16-times higher protein levels, respectively (*p* < 0.05, *versus* untreated control). While for caspase 9, a similar pattern was observed but to a lesser extent.

### 3.6 RT-qPCR results of PI3K/AKT/mTOR, KRAS/BRAF/MAPK pathways and VEGF ELISA

**FIGURE 7:**
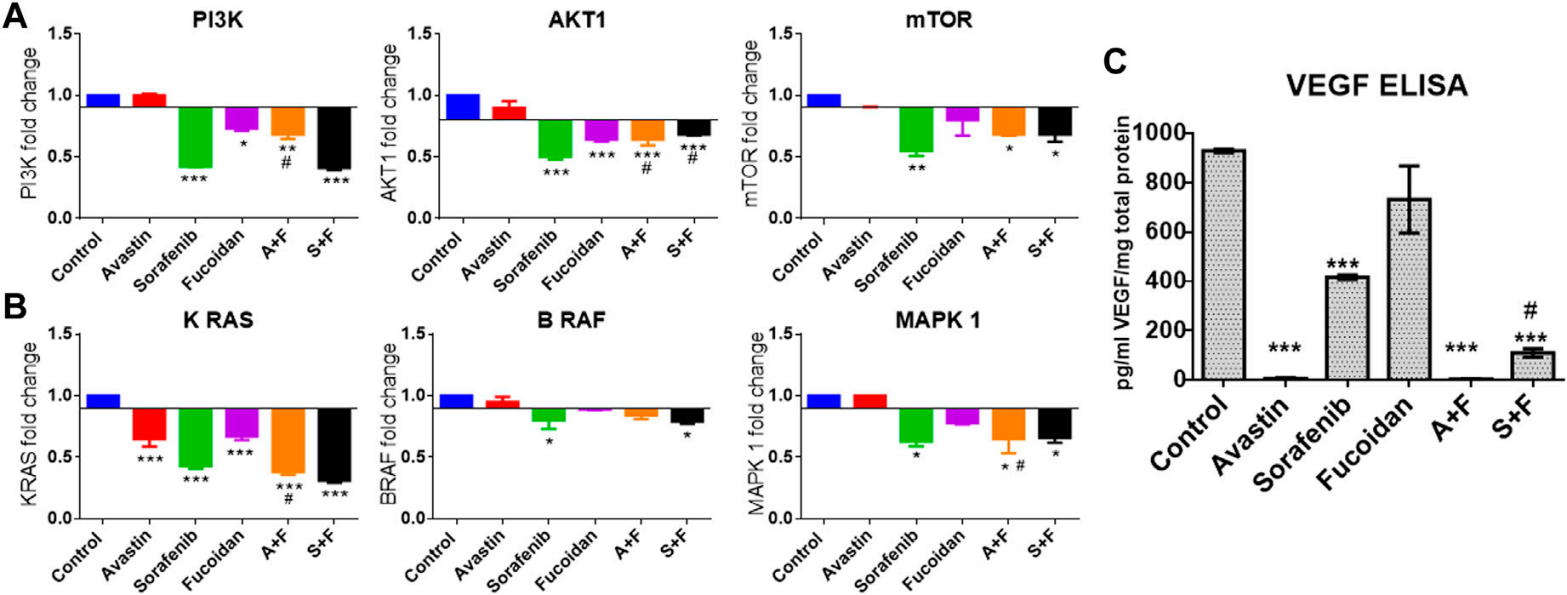
Effect of the drug combinations on VEGF and its downstream signaling molecules. **(A, B)** RT-qPCR results showing the fold change in mRNA levels (calculated using 2-^ΔΔ^CT method) of the key angiogenic genes: PI3K, AKT, mTOR, KRAS, BRAF and MAPK in HUH-7 cells. Sorafenib, A + F and S + F significantly reduced the expression of the oncogenic PI3K/AKT/mTOR and the KRAS/BRAF/MAPK pathways. The most affected were PI3K and KRAS with a decreased mRNA levels of 1.5 and 2.4 folds with A + F or 2.6 and 3.2 folds with S + F, respectively. **(C)** VEGF ELISA results showing a significant reduction in intracellular VEGF levels. Cells treated with Avastin and A + F showed the strongest reduction followed by S + F, sorafenib and fucoidan. *p < 0.05, **p < 0.005, ***p < 0.001 *versus* untreated control or ^#^ *p* < 0.05 compared to monotherapy, one-way ANOVA with Tukey’s *post hoc*.

Next, we evaluated the effect of the drug alone and in combinations on the key pro-angiogenic pathways: VEGF (using ELISA) and its downstream signaling molecules, PI3K/AKT/mTOR and KRAS/BRAF/MAPK pathways *via* mRNA expression levels by RT-qPCR. Fucoidan, sorafenib, A + F and S + F significantly reduced the expression of the pro-angiogenic PI3K/AKT/mTOR and KRAS/BRAF/MAPK pathways by up to 3 folds (*p* < 0.05 *versus* untreated control). The most affected were PI3K and KRAS with a significantly decreased mRNA levels of 1.5 and 2.4 folds with A + F or 2.6 and 3.2 folds with S + F, respectively (*p* < 0.05 *versus* untreated control) (see Figure 7).

### 3.7 Diethyl nitrosamine (DEN) HCC rat model

**FIGURE 8:**
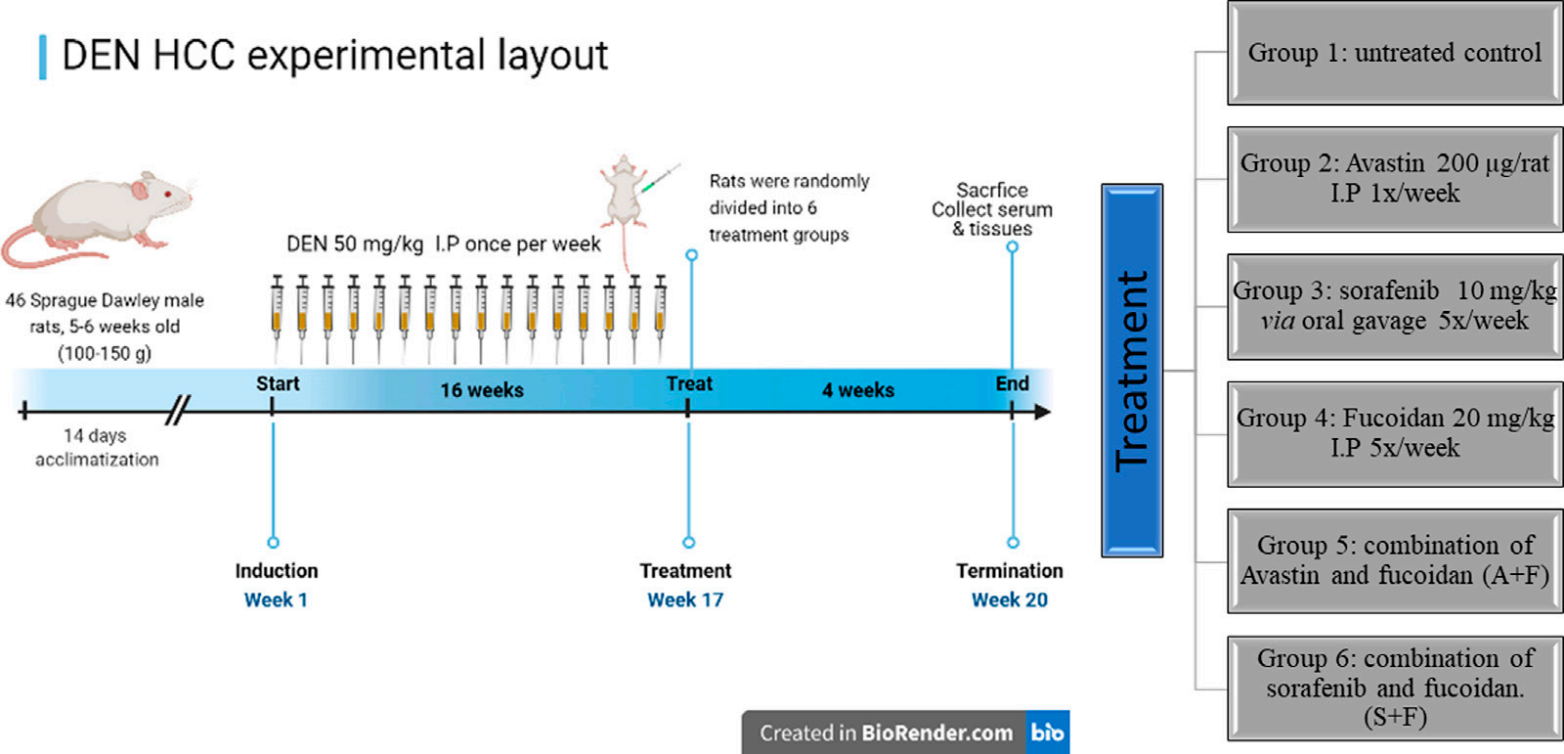
A diagram depicting the *in vivo* experimental timeline used to develop the DEN HCC rat model as well as the different treatment groups (created in Biorender.com).

As previously described in the methodology, rats were given weekly injections of DEN for 16 weeks then treated for a month with the different drugs and respective combinations. At the end of the experiment, rats were sacrificed, and blood and tissue samples were collected (summary of experimental timeline is shown in Figure 8).

**FIGURE 9:**
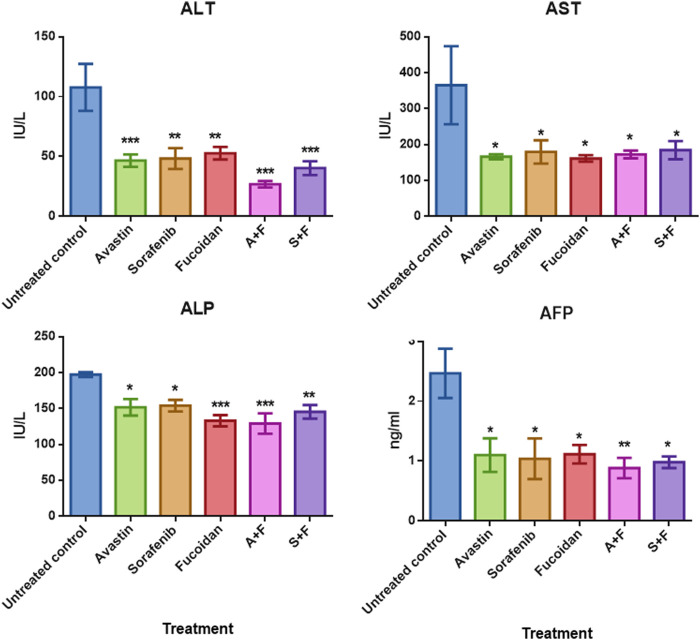
The effect of the different treatments on the levels of serum transaminases, ALP and AFP in DEN-HCC rat models. All drug treatments significantly reduced the levels of ALT, AST, ALP and AFP with no significant difference detected amongst the treatment groups. n = 3–5/group, *p < 0.05, **p < 0.005, ***p < 0.001, one-way-ANOVA with Tukey’s *post hoc versus* untreated control.

Assessment of liver function was done by measuring aspartate aminotransferase (AST), alanine aminotransferase (ALT) and alkaline phosphatase (ALP) in the rats’ serum samples using commercially available colorimetric kits. While Alpha fetoprotein (AFP), a key marker of tumour burden and prognosis (Morse et al., 2019), was assessed using ELISA. Results (Figure 9) showed that the untreated controls had the highest level of ALT, AST, ALP and AFP indicating liver damage and high tumour burden. While all treated groups showed significantly lower levels of these biomarkers compared to the untreated control with no significant difference amongst the different treatments.

### 3.8 Histological analysis of the liver tissues and other organs of DEN HCC rat model

**FIGURE 10:**
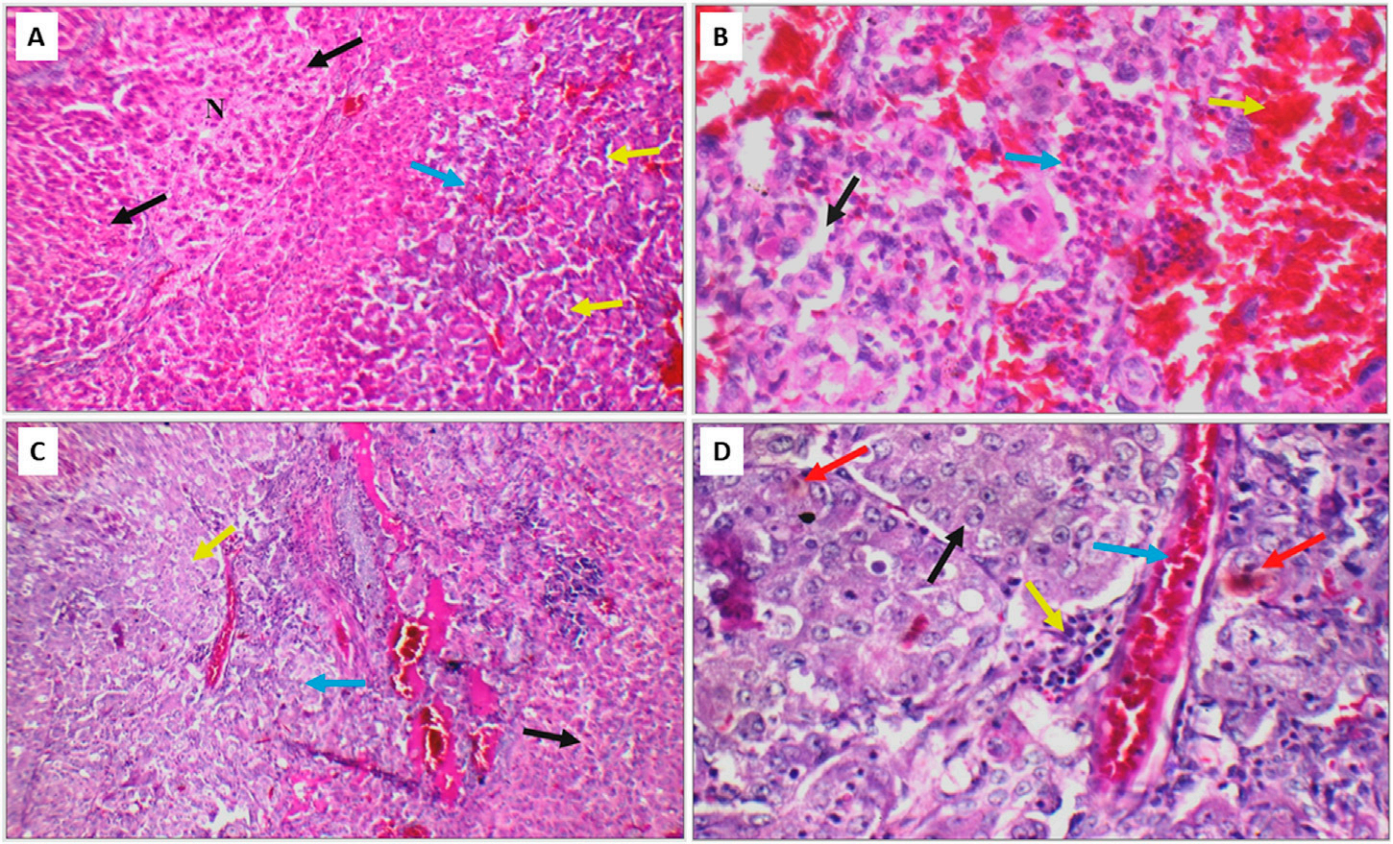
**(A)** DEN-treated positive control liver sections showing area of cirrhosis with well-defined nodules (black arrow), and area of HCC (blue arrow) with acinar formation (yellow arrow) (H&E X 200). **(B)** Another high-power view of the same positive control liver section showing HCC with markedly pleomorphic cells and acinar formation (black arrow), marked areas of hemorrhage (yellow arrow) and inflammatory infiltrate (blue arrow) (H&E X 400). **(C)** Liver section of another positive control showing areas of cirrhosis (black arrow), and area of HCC (blue arrow) with trabecular pattern (yellow arrow) (H&E X 200). **(D)** High power view of the positive control shown in C revealing trabecular HCC with markedly pleomorphic cells and prominent nuclei (black arrow), markedly congested blood vessels (blue arrow), mild inflammatory infiltrate (yellow arrow), and scattered apoptosis (red arrow) (H&E X 400).

**FIGURE 11:**
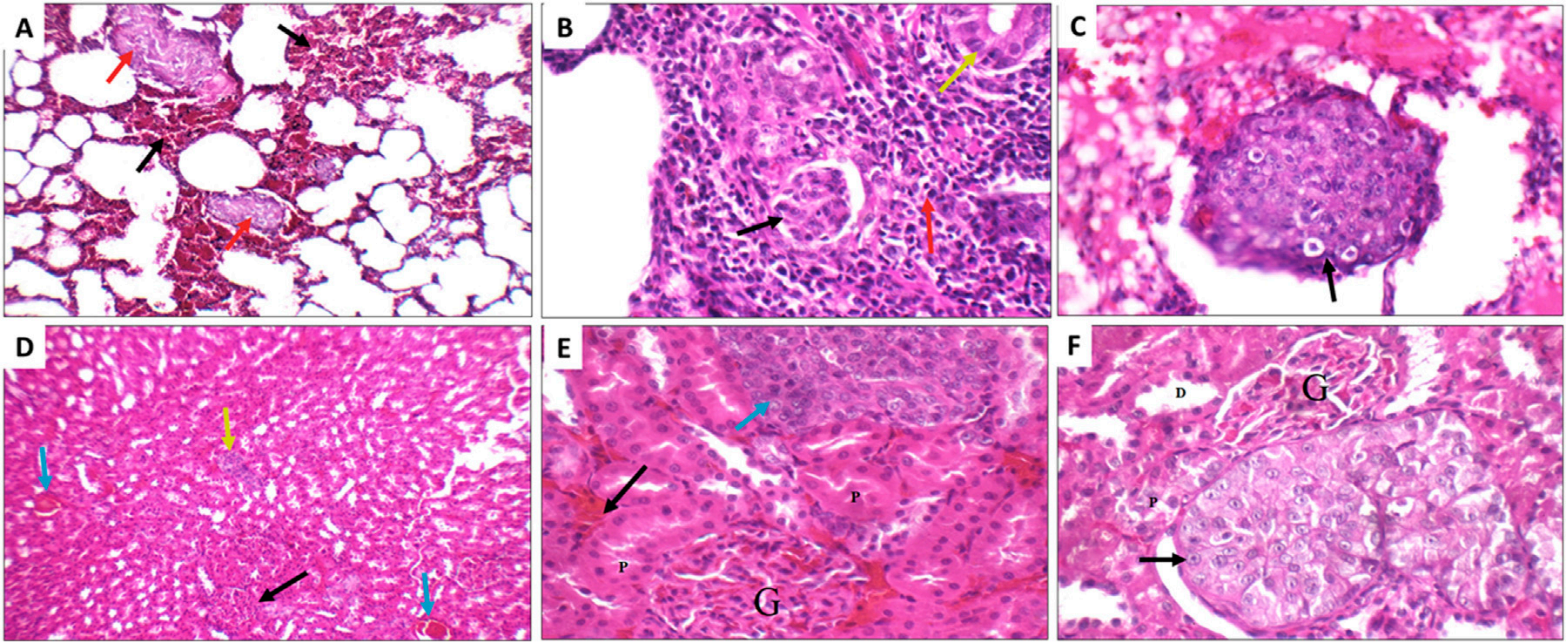
Metastatic nodules detected in the lungs **(A–C)** and kidneys **(D–F)** of the DEN rats from the different treatment groups. **(A)** Positive control lung showing marked interstitial hemorrhage (black arrow) with small metastatic nodules (red arrow) (H&E X 200). **(B)** Sorafenib treated rat showing metastatic adenocarcinoma (yellow arrow), with marked inflammatory infiltrate (red arrow), and scattered tumour emboli (black arrow) (H&E X 400. **(C)** Fucoidan treated rats showing vascular tumour emboli (black arrow) (H&E X 400). **(D)** Sorafenib treated rat kidney showing average glomeruli (black arrow), areas of interstitial hemorrhage (blue arrow), and metastatic nodule (yellow arrow) (H&E X 200). **(E)** Avastin treated rat showing average glomeruli (G), and average proximal tubules (P), areas of interstitial hemorrhage (black arrow), and metastatic nodule (blue arrow) (H&E X 400). **(F)** Avastin treated rat showing average glomeruli (G), distal tubules (D) and average proximal tubules (P), and metastatic nodule of malignant cells with prominent nucleoli (black arrow) (H&E X 400).

Next, the histopathological changes induced by DEN was evaluated using hematoxylin and eosin staining (H&E) done on livers, kidneys and lungs tissue sections. All DEN treated rats developed at least cirrhosis with 25 out of 35 rats progressing to HCC, therefore, the success rate of this model was 71.4%. Both acinar (grade I) (Figures 10A, B) and trabecular (grade II) (Figures 10C, D) HCC nodules were observed within the liver tissues with acinar HCC nodules being the most common. Metastatic tumour masses were also observed in the lungs and kidneys of DEN-treated rats regardless of the treatment given (Figure 11).

**FIGURE 12:**
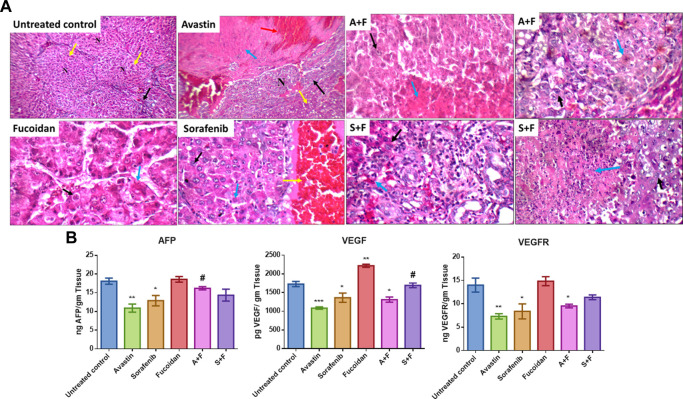
**(A)** Liver sections of DEN treated rats after treatment with the various drugs and their respective combination. Positive control showing fibrotic portal tract (black arrow), with well-defined nodules (yellow arrow, N) (H&E X 200). Avastin treated group showing incomplete nodules and mild micro-vesicular steatosis (yellow arrow) and large nodule of HCC (black arrow) with marked areas of necrosis (blue arrow) and hemorrhage (red arrow) (H&E X 200). Sorafenib treated group showing acinar HCC composed of mildly pleomorphic cells with prominent nucleoli (black arrow) and scattered apoptotic cells (blue arrow) and marked areas of hemorrhage (yellow arrow) (H&E X 400). Fucoidan treated group showing trabecular HCC composed of markedly pleomorphic cells with prominent nucleoli (black arrow) with marked apoptosis (blue arrow) (H&E X 400). A + F slide showing trabecular HCC composed of mildly pleomorphic cells with prominent nucleoli (black arrow) with marked areas of necrosis (blue arrow) (H&E X 400) a second slide of A + F showing large nodule of HCC composed of mildly pleomorphic cells with prominent nucleoli (black arrow) with marked apoptosis (blue arrow) (H&E X 400). S + F showing large nodule of acinar HCC composed of markedly pleomorphic cells (black arrow) with large areas of necrosis (blue arrow) (H&E X 400) a second slide of S + F showing large nodule of acinar HCC composed of mildly pleomorphic cells (black arrow) with marked apoptosis (blue arrow) (H&E X 400). **(B)** Levels of VEGF, VEGFR and AFP in liver tissues as detected with ELISA. Avastin and sorafenib monotherapies significantly decreased the levels of AFP while fucoidan was similar to the untreated control. Although the combination therapies decreased the levels of AFP, the results were not statistically significant compared to the untreated control. VEGF and its receptor VEGFR were significantly reduced by Avastin, sorafenib and A + F compared to the untreated control. While S + F also markedly reduced their levels, but the results were statistically insignificant. For VEGF, fucoidan alone slightly but significantly increased its levels compared to the control unlike VEGFR as fucoidan had very similar levels to the control. n = 3–5, *p < 0.05, **p < 0.005, ***p < 0.001, one-way-ANOVA with Tukey’s *post hoc versus* untreated control, #*p* < 0.05 *versus* monotherapies.

Upon evaluation of the tumour sections of the untreated control, we observed large nodules of HCC with markedly pleomorphic cells accompanied by prominent nucleoli, scattered apoptosis, mild inflammatory infiltrate, mildly congested blood vessels and marked areas of hemorrhage and inflammatory infiltrate. While smaller tumour nodules as well as marked areas of apoptosis and necrosis were observed in the tumour nodules of rats treated with the different drugs with more prominent apoptotic and necrotic areas observed in the combination treated groups (Figure 12A).

### 3.9 Measuring the levels of VEGF, VEGFR and AFP in liver tissues using ELISA

Liver tissues extracted from DEN HCC model were analyzed using ELISA for the levels of AFP, VEGF, and VEGFR.

AFP, a key marker of liver tumour burden (Morse et al., 2019), was assessed in the different treatment groups. Results (Figure 12B) revealed that Avastin and sorafenib monotherapies significantly decreased the levels of AFP while fucoidan was similar to the untreated control. Although the combination therapies decreased the levels of AFP, the results were not statistically significant compared to the untreated control.

The key angiogenic protein VEGF and its receptor VEGFR were also assessed (Figure 12B). In both instances Avastin, sorafenib and A + F significantly reduced their levels compared to the untreated control. While S + F also markedly reduced their levels, but the results were insignificant. Surprisingly, for VEGF, fucoidan alone slightly but significantly increased its levels compared to the control unlike VEGFR as fucoidan had very similar levels to the control.

### 3.10 Immunohistochemistry analysis of caspase 3, CD34 and Ki67

Liver tissues were stained for the key apoptotic executioner caspase 3, one of the main proliferation markers (Ki67) (Morse et al., 2019) and CD34, the highly sensitive marker for endothelial cells and is generally used as a marker of angiogenesis in tumours (Vieira et al., 2005).

**FIGURE 13:**
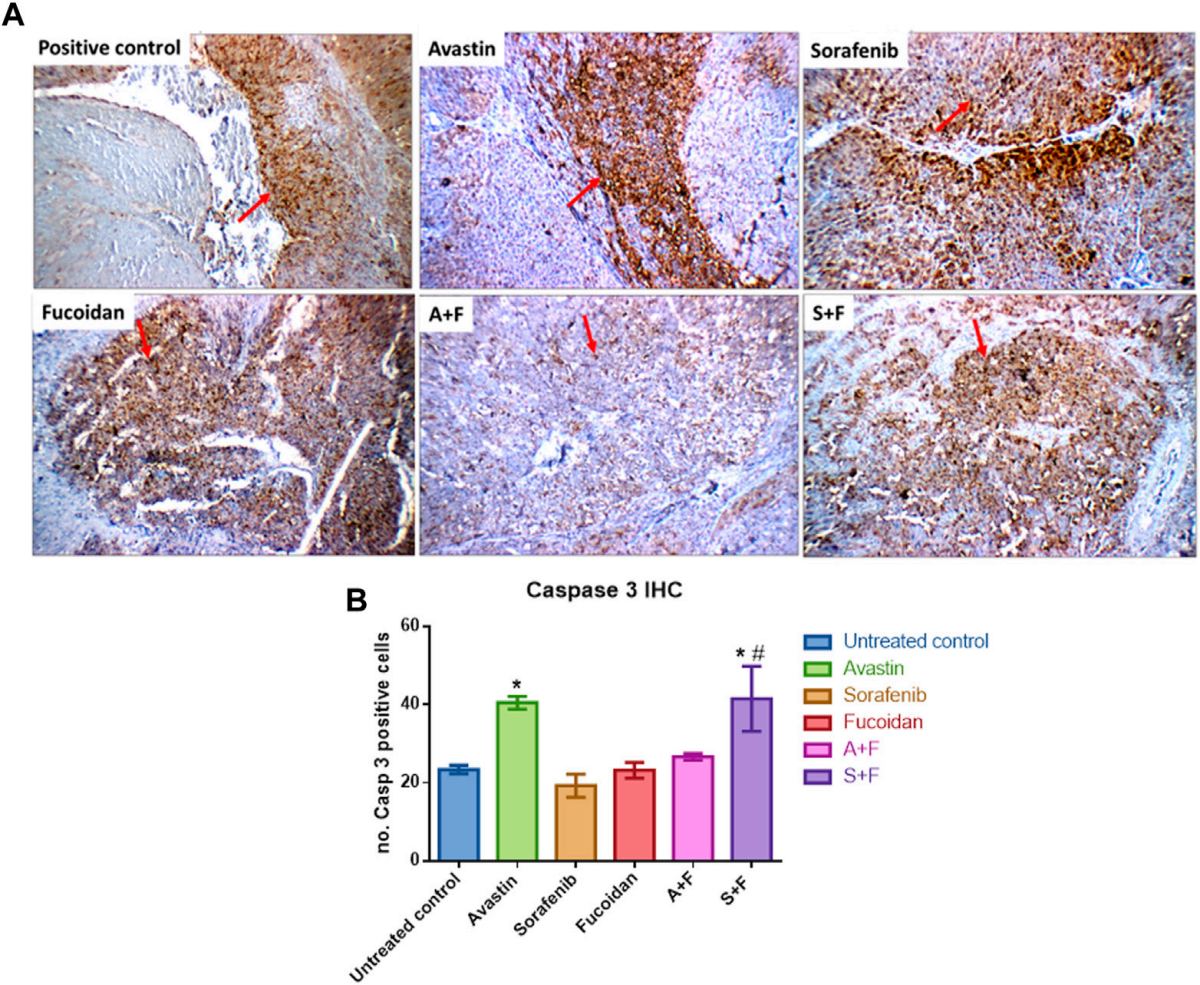
Immunohistochemistry analysis of caspase 3 (red arrows) in tumor nodules within liver tissues. **(A)** Photomicrographs of livers extracted from rats treated with the different drugs. Positive control liver view showing moderate cytoplasmic reactivity (++) for caspase 3. Avastin treated liver tissue showing marked cytoplasmic reactivity (+++). Sorafenib treated liver showing moderate cytoplasmic reactivity (++). Fucoidan and A + F showing moderate cytoplasmic reactivity (++) for caspase-3 in tumor tissue and finally S + F liver showing marked cytoplasmic reactivity (+++) for caspase-3 in tumor tissue. (Caspase 3 immunostaining (red arrows) and Hematoxylin counterstain, x 200). **(B)** Number of caspase 3 positive cells showing that the combination of S + F was significantly better than sorafenib alone, but an opposite pattern was observed for Avastin. Avastin monotherapy showed significantly higher levels of caspase 3. (n = 3–5, *p < 0.05 *versus* untreated control, #*p* < 0.05 *versus* monotherapies, one-way-ANOVA with Tukey’s *post hoc*).

Caspase 3 reactivity was predominantly cytoplasmic with some nuclear staining. The interpretation of the results considered both the staining intensity (Figure 13A) and the percentage of positive cells (Figure 13B). The reactivity was classified as: negative (0), weak (+), moderate (++) or marked (+++). In the untreated control, liver showed moderate cytoplasmic reactivity (++) for caspase-3 in cirrhotic and tumor nodules. The most significant findings were observed in rats treated with Avastin and S + F, liver tissues showed moderate cytoplasmic reactivity (++) for caspase-3 in liver tissue and marked reactivity (+++) in tumor nodules. While Avastin, fucoidan and A + F were very similar to the untreated control with moderate cytoplasmic reactivity (++) for caspase-3 in tumor nodules and sorafenib alone showing weak reactivity (+) in tumor nodules. The results indicate that the combination of S + F was significantly better than sorafenib alone, but an opposite pattern was observed for Avastin (Figure 13B).

**FIGURE 14:**
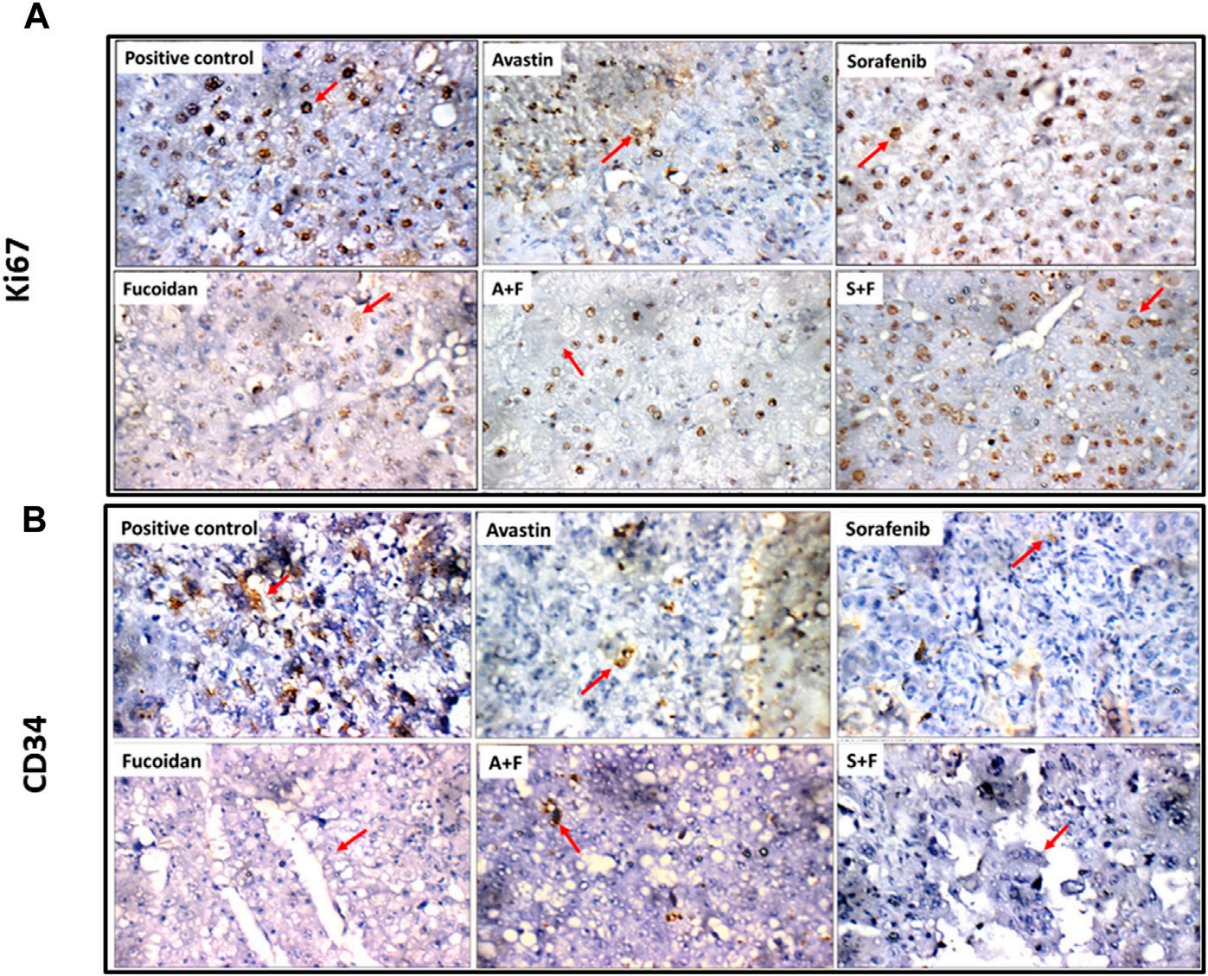
**(A)** Photomicrographs showing immunohistochemical staining for Ki67 in tumor nodules (red arrows) within liver tissues extracted from rats treated with the different drugs. Ki67 positivity was evaluated according to percentage of positive cells into four degree: (-) < 24%, (+) 25%–50% (Isolated), (++) 51%–74% (Focal), (+++) > 75% (Diffuse) (Mocanu et al., 2012). Positive control showing focal reactivity (++), Avastin showing negative reactivity (-), Sorafenib showing isolated reactivity (+) while fucoidan showing negative reactivity (-) for Ki67. A + F showing negative reactivity (-) for Ki67 and S + F showing isolated reactivity (+) (Ki67 immunostaining (red arrows), x 400). **(B)** Photomicrographs showing immunohistochemical staining for CD34 in endothelial cells in tumor nodules (red arrows) within liver tissues extracted from rats treated with the different drugs. The number of positive cells for CD34 staining was evaluated as follows: (0 = 0%, 1 = 1–25%, 2 = 26–50%, 3 = 51–75%, and 4 = 76–100% of cells) as described by (Kamat et al., 2006). Positive control showing moderate reactivity (2) for CD34 in tumor nodules. Avastin and sorafenib showing weak reactivity (1) while fucoidan showing negative reactivity (0) for CD34 in tumor nodules. A + F showing weak reactivity (+) while S + F showing negative reactivity (0) for CD34 in tumor nodules (red arrow) (CD34 immunostaining (red arrows), x 400).

Ki67 positivity was evaluated according to percentage of positive cells into four degree: (-) < 24%, (+) 25%–50% (Isolated), (++) 51%–74% (Focal) or (+++) > 75% (Diffuse) (Mocanu et al., 2012). In the untreated control, liver showed focal reactivity (++) for Ki67 in cirrhotic and tumor nodules. This pattern was reduced in all treatment groups to various degrees. In Avastin treated groups, liver tissues showed isolated reactivity (+) for Ki67 in cirrhotic nodules and negative reactivity (-) in tumor nodules. For sorafenib, liver showed isolated reactivity (+) for Ki67 in cirrhotic and tumor nodules. In fucoidan, negative reactivity (-) for Ki67 in tumor nodules was detected. In the combination groups, A + F liver showed isolated reactivity (+) for Ki67 in tumor nodules, while S + F liver showed negative reactivity (-) for Ki67 in cirrhotic nodules, and isolated reactivity (+) in tumor nodules (as shown in Figure 14A).

Finally, for CD34 staining, the number of positive cells was evaluated as follows: (0 = 0%, 1 = 1–25%, 2 = 26–50%, 3 = 51–75%, and 4 = 76–100% of cells) as described by (Kamat et al., 2006). Untreated control liver showed moderate reactivity (2) for CD34 in cirrhotic and tumor nodules while in the treated groups weak or negative reactivity was detected. For Avastin and sorafenib weak reactivity (1) for CD34 was observed in cirrhotic and tumor nodules. Fucoidan showed negative reactivity (0) for CD34 in tumor nodules. For the combinations, A + F liver showed negative reactivity (0) for CD34 in cirrhotic nodules, and weak reactivity (+) in tumor nodules while S + F showed an opposite pattern with weak reactivity (+) for CD34 in cirrhotic nodules, and negative reactivity (0) in tumor nodules (as shown in Figure 14B).

## 4 Discussion

Interestingly, 90% of cancer patients have reported the use of complementary and alternative medicines during their treatment, with 70% of patients not discussing the use of such agents with their healthcare professionals (Bahall, 2017; Wu et al., 2022). Being an herbal medicinal product, fucoidan is a particularly popular agent that is readily available as an over the counter (OTC) herbal supplement in many countries around the globe with many cancer patients reporting its use (Wu et al., 2022). Moreover, fucoidan have attracted a lot of attention in recent years with a 100,000,000$ worth of fucoidan related supplements being produced annually (Tsai et al., 2017). This highlights the importance of our research that is studying the combination of fucoidan, which has a proven anti-cancer potential as reported in many preclinical studies (Jin et al., 2022), with 2 clinically approved anti-angiogenic agents (Avastin and sorafenib) in HCC.

HCC is a highly vascular tumour and thus represents an exciting target for the development of anti-angiogenic drugs. Nonetheless, to date, sorafenib and Avastin are the only anti-angiogenic agents that are clinically used in the treatment of HCC. This highlights, the challenges of targeting this key process for the treatment of HCC.

Fucoidan has been investigated before in combination with Avastin but for the treatment of exudative age-related macular degeneration, results showed an *in vitro* reduction in the expression of VEGF (Dithmer et al., 2014). While the combination of fucoidan with sorafenib has been recently reported *in vitro* in a sorafenib resistant cell line (HepG2-SR) and *in vivo* using tumour xenograft in nude mice (Luo et al., 2022). The results of this study focused on the epidermal growth factor receptor (EGFR) pathway and showed that fucoidan could help overcome sorafenib resistance in HCC *via* binding to EGFR (Luo et al., 2022). Nonetheless, despite these previously reported promising findings, our research explores a poorly investigated area of research which is the interaction between fucoidan and the key angiogenic pathway especially in combination with sorafenib and Avastin in HCC.

Published reports have revealed contradicting data about the interaction of fucoidan with the angiogenic pathways, specifically with the key angiogenic promotor: the vascular endothelial growth factor (VEGF). It was reported that fucoidan inhibited binding of VEGF to its receptor (VEGFR) (Koyanagi et al., 2003) and reduced VEGF expression both *in vitro* and *in vivo* in mouse breast cancer cells (Xue et al., 2012). On the contrary, another study reported that fucoidan had a potent growth inhibitory effect on HCC tumorigenesis without interfering with angiogenesis and VEGF expression both *in vitro* and *in vivo* (Zhu et al., 2013). These discrepancies have been attributed to the differences in the molecular weight and chemical structures (such as the degree of sulfation) of the tested fucoidans (Kwak, 2014; Atashrazm et al., 2015).

First, we investigated the combination of fucoidan with sorafenib and Avastin *in vitro* on a human HCC cell line; HUH-7. Results revealed a strong synergistic interaction between fucoidan, and the 2 anti-angiogenic drugs as measured with the MTT assay. Next, we did several functional assays to assess the effect of the combination therapy on cancer cell motility, induction of apoptosis and cell cycle progression. The results were not as clear as the cell viability assay, even though in most of these assays the drugs and their combinations were significantly better than the untreated control, the differences between the monotherapies and the combination therapies were not always as striking or statistically significant. In the scratch wound assay, results revealed that sorafenib and the combination therapies significantly inhibited wound healing as a significantly smaller % wound closure was observed (50%–70%) *versus* untreated control (91%–100%). Non-etheless, A + F showed slightly better but insignificant difference to Avastin alone while sorafenib and S + F showed very similar patterns. However, in the apoptosis Annexin V/PI assay, A + F showed significantly less viable cells as well as higher percentage of cells in early apoptosis and necrosis compared to Avastin monotherapy which was very similar to the control. Both sorafenib and S + F were significantly better than the untreated control, but both showed very similar patterns to each other. In the cell cycle analysis, both Avastin and sorafenib monotherapies were similar to the untreated control but again fucoidan appeared to direct the cells towards a G2/M arrest. S + F and A + F had a significantly higher percentage of cells in the G2/M phase as compared to their monotherapies.

Next, we embarked on searching for the potential mechanistic interactions between the drugs under investigation. We evaluated the VEGFR pathway and two of its main downstream signaling cascades: the PI3K/AKT/mTOR and the RAS/RAF/MAPK pathways (Dimri and Satyanarayana, 2020). These pathways are commonly dysregulated in HCC and are downstream from many growth factor receptors (e.g. VEGFR, EGFR, fibroblast growth factor receptor (FGFR) and the platelet-derived growth factor receptor (PDGF)) (Dimri and Satyanarayana, 2020). Moreover, the hyperactivation of the PI3K/AKT/mTOR and the RAS/RAF/ERK/MAPK pathways and the overexpression of growth factors (e.g., fibroblast growth factor (FGF)) combined with the overactivation of processes like angiogenesis and epithelial to mesenchymal transition (EMT) are the main culprits in the tumorigeneses of HCC (Lamarca et al., 2016). Therefore, it was crucial to elucidate the effect of our drug combinations on these pathways.

VEGF ELISA done on cell lysates revealed that Avastin, as expected, completely depletes VEGF and similarly A + F showed similar findings. These results are very encouraging because the interaction of fucoidan with VEGF has been a point of controversary in the literature as previously mentioned. Based on the findings presented herein, it is important to highlight that a) fucoidan alone deceased the levels of VEGF (although the results were statistically insignificant compared to the untreated control). (b) It did not interfere with the binding of Avastin to VEGF as similar to Avastin alone, A + F significantly reduced VEGF levels and (c) it strongly potentiated the effect of sorafenib as S + F treated cells had significantly lower VEGF levels. For the PI3K/AKT/mTOR axis, Avastin alone did not induce significant changes compared to the untreated control while A + F significantly reduced the mRNA levels of all three genes. In both sorafenib and S + F groups, the PI3K/AKT/mTOR mRNA levels were also reduced compared to the untreated control with no statistically significant difference between sorafenib and S + F for the PI3K and mTOR.

In summary, it appears that fucoidan potentiated the effect of Avastin in inhibiting the PI3K/AKT/mTOR and the RAS/RAF/MAPK pathways but neither potentiated nor significantly negated the effect of sorafenib on these pathways.

Finally, we evaluated the effect of the drugs on three key apoptosis related proteins, caspase 3, 8, and 9. Fucoidan significantly potentiated the levels of caspase 3 and 8 when combined with the anti-angiogenic drugs (A + F and S + F) either when compared to the untreated control or the monotherapies. While for caspase 9, all drugs and combinations (except Avastin) increased its level but to a lesser extent compared to caspase 3 and 8. Fucoidan was previously reported to induce cell death in HCC cell lines. In a recent study by (Duan et al., 2020), fucoidan induced cell death in LM3 HCC cell line when measured using the Annexin V/PI assay where a higher percentage of cells were detected in early and late apoptosis phases while our study showed cells to be mostly in the necrotic phase. They also reported that this was mediated *via* the promotion of the phosphorylation of p38 MAPK, the reduction of ERK, PI3K and AKT phosphorylation as well as the activation of caspase 3, 8, and 9. These findings are similar to ours except for MAPK which we reported the suppression of its mRNA levels by fucoidan and the combination therapy while (Duan et al., 2020) reported their findings on a protein level.

*In vivo*, we established the DEN HCC tumour model by a weekly injection of DEN for 16 weeks (Schiffer et al., 2005) and then rats were treated with the drugs and combinations for 4 extra weeks. Generally, we noticed a lot of variations amongst the rats of the same group, a major drawback for carcinogen induced cancer models along with undefined genetic backgrounds detected in the tumours (Macek Jilkova et al., 2019). Microscopically, areas of marked apoptosis and necrosis were observed within the tumour nodules of the treated mice with more areas detected in the combination therapies. Results of the liver function tests (ALT, AST, and ALP) and liver tumour marker (AFP) revealed a significant improvement in all treatment groups but no clear advantage of fucoidan was detected. Surprisingly, tissue levels of AFP, VEGF and VEGFR were slightly elevated with fucoidan alone, but the results were only statistically significant for VEGF. For the combination therapies, the results were always significantly less than the untreated control but when compared to monotherapies variable patterns were detected. For AFP, A + F and S + F showed slightly higher levels but only A + F was significantly higher than Avastin. For VEGF, no significant difference was observed in the Avastin *versus* A + F group unlike the S + F group which was slightly higher than sorafenib. Finally, in VEGFR, no significant difference was observed between the monotherapies and the combination therapies. Finally, we performed IHC to assess the levels of the apoptotic marker caspase 3, the proliferation marker Ki67 and the marker for angiogenesis CD34. We only quantitively assessed caspase 3 as it has the clearest and strongest staining. Results revealed that only Avastin and S + F significantly increased the levels of caspase 3 with fucoidan potentiating the effect of sorafenib but antagonizing the effect of Avastin. Unlike for Ki67 and CD34 as the staining was semiquantitative; analysis revealed weak or negative reactivity in all treatment groups unlike the untreated control. In summary, the *in vivo* results appear to show neither a clear benefit of fucoidan nor a strong antagonistic effect. We found limited data reporting the use of fucoidan in DEN HCC. In a study by (Suresh et al., 2013) they reported that fucoidan inhibited the metabolic activation of DEN and consequently protected against DEN-induced hepatocarcinogenesis (Suresh et al., 2013; Jin et al., 2022).

## 5 Summary and conclusions

In summary, we believe that our findings emphasize the importance of studying the combination of commonly used herbal medicinal products with clinically approved drugs. Numerous patients are particularly interested in consuming fucoidan in hope of potentiating the effect of anti-cancer drugs. Our research highlighted a promising chemomodulatory effect of fucoidan when combined with sorafenib and Avastin although further investigations are required to elucidate potential beneficial or adversary interactions between the tested therapies.

## Data Availability

The original contributions presented in the study are included in the article/supplementary material, further inquiries can be directed to the corresponding author.
